# Supplementary material for: Q Fever Endocarditis in Iran
Source: Sci Rep. 2019 Oct 24;9:15276. doi: 10.1038/s41598-019-51600-3 (PMC6813299; doi:10.1038/s41598-019-51600-3)
Supplement: Supplementary file 1 — Supplementary Table 1 [file 41598_2019_51600_MOESM1_ESM.docx]

Q Fever Endocarditis in Iran

Running title: Q fever Endocarditis in Iran

Pardis Moradnejad^1^, Saber Esmaeili^2,3,4^, Majid Maleki^1^, Anita Sadeghpour^1^, Monireh Kamali^1^, Mahdi Rohani^2,5^, Ahmad Ghasemi^2,3,4^, Fahimeh Bagheri Amiri^2^, Hamid Reza Pasha^1^, Shabnam Boudagh^6^, Hooman Bakhshandeh Abkenar^6^, Nasim Naderi^6^, Behshid Ghadrdoost^6^, Sara Lotfian^6^, Seyed Ali Dehghan Menshadi^7^, Ehsan Mostafavi^2,3*^

1. Echocardiography Research center, Rajaie Cardiovascular Medical and Research Center, Iran University of Medical Sciences, Tehran, Iran.
2. National Reference Laboratory for Plague, Tularemia and Q fever, Research Centre for Emerging and Reemerging Infectious Diseases, Pasteur Institute of Iran, Akanlu, Kabudar Ahang, Iran.
3. Department of Epidemiology and Biostatistics, Research Centre for Emerging and Reemerging infectious diseases, Pasteur Institute of Iran, Tehran, Iran
4. Department of Bacteriology, Faculty of Medical Sciences, Tarbiat Modares University, Tehran, Iran.
5. Department of Bacteriology, Pasteur Institute of Iran, Tehran, Iran.
6. Rajaie Cardiovascular Medical and Research Center, Iran University of Medical Sciences, Tehran, Iran.
7. Department of Infectious Diseases and Tropical Medicine, Tehran University of Medical Sciences, Tehran, Iran

**Corresponding Author:** Dr Ehsan Mostafavi No. 69, Pasteur Ave., Department of Epidemiology and Biostatistics, Research Centre for Emerging and Reemerging Infectious Diseases, Pasteur Institute of Iran, Postal Code: 1316943551, Tehran, Iran, Telefax: +98-21-66496448, Email: [mostafavi@pasteur.ac.ir](mailto:mostafavi@pasteur.ac.ir), [mostafaviehsan@gmail.com](mailto:mostafaviehsan@gmail.com)

Supplementary Table 1. Diagnostic guideline for Q fever endocarditis proposed by Raoult criteria^25*^.

| Q fever endocarditis |
| --- |
| 1. **Definite criteria**   Positive culture, PCR, or immunochemistry of a cardiac valve   1. **Major criteria**   Microbiology: positive culture or PCR of the blood or an emboli or serology with IgG I antibodies ≥6,400 Evidence of endocardial involvement:  Echocardiogram positive for IE: oscillating intra-cardiac mass on valve or supporting structure, in the path of regurgitant jets, or on implanted material in the absence of an alternative anatomic explanation; or abscess; or new partial dehiscence of prosthetic vale; or new valvular regurgitation (worsening or changing of pre-existent murmur not sufficient)  PET scan showing a specific valve fixation and mycotic aneurysm   1. **Minor Criteria**   Predisposing heart condition (known or found on echocardiograph)  Fever, temperature >38°C  Vascular phenomena, major arterial emboli, septic pulmonary infarcts, mycotic aneurysm (see at PET scan), intracranial hemorrhage, conjunctival hemorrhages, and Janeway lesions Immunologic phenomena: glomerulonephritis, Osle nodes, Roth spots, or rheumatoid factor  Serologic evidence: IgG I antibodies ≥800 <6,400  Diagnosis definite   1. 1A criterion 2. 2B criterion 3. 1B and 3C criterion   Diagnosis possible   1. 1B criterion, 2C criteria (including microbiology evidence, and cardiac predisposition) 2. 3C criteria (including positive serology, and cardiac predisposition) |
| Q fever vascular infection |
| 1. **Definite criteria**   Positive culture, PCR, or immunochemistry of an arterial sample (prosthesis or aneurysm) or a periarterial abscess or a spondylodiscitis linked to aorta   1. **Major criteria**   Microbiology: positive culture or PCR of the blood or an emboli or serology with IgG I antibodies ≥6,400 Evidence of vascular involvement  CT scan: aneurysm or vascular prosthesis+periarterial abscess, fistula, or spondylodiscitis  PET scan: specific fixation on an aneurysm or vascular prosthesis   1. **Minor criteria**   Serological IgG I ≥800 <6,400  Fever, temperature >38°C  Emboli Underlying vascular predisposition (aneurysm or vascular prosthesis)  Diagnosis definite   1. 1A criterion 2. 2B criterion 3. 1B and 2C criterion (including microbiology findings and vascular predisposition)   Diagnosis possible  Vascular predisposition, serological evidence and fever or emboli |

IE, infective endocarditis; PET, positron emission tomography; IFA, immunofluorescence assay; CT, computed tomography.

*This table is completely adapted from the reference number.
