# Supplementary material for: Q Fever Endocarditis in Iran
Source: Sci Rep. 2019 Oct 24;9:15276. doi: 10.1038/s41598-019-51600-3 (PMC6813299; doi:10.1038/s41598-019-51600-3)
Supplement: Supplementary file 2 — Revised manuscript by Track change [file 41598_2019_51600_MOESM2_ESM.docx]

Q Fever Endocarditis in Iran

Running title: Q fever Endocarditis in Iran

Pardis Moradnejad^1^, Saber Esmaeili^2,3,4^, Majid Maleki^1^, Anita Sadeghpour^1^, Monireh Kamali^1^, Mahdi Rohani^2,5^, Ahmad Ghasemi^2,3,4^, Fahimeh Bagheri Amiri^2^, Hamid Reza Pasha^1^, Shabnam Boudagh^6^, Hooman Bakhshandeh Abkenar^6^, Nasim Naderi^6^, Behshid Ghadrdoost^6^, Sara Lotfian^6^, Seyed Ali Dehghan Menshadi^7^, Ehsan Mostafavi^2,3*^

1. Echocardiography Research center, Rajaie Cardiovascular Medical and Research Center, Iran University of Medical Sciences, Tehran, Iran.
2. National Reference Laboratory for Plague, Tularemia and Q fever, Research Centre for Emerging and Reemerging Infectious Diseases, Pasteur Institute of Iran, Akanlu, Kabudar Ahang, Iran.
3. Department of Epidemiology and Biostatistics, Research Centre for Emerging and Reemerging infectious diseases, Pasteur Institute of Iran, Tehran, Iran
4. Department of Bacteriology, Faculty of Medical Sciences, Tarbiat Modares University, Tehran, Iran.
5. Department of Bacteriology, Pasteur Institute of Iran, Tehran, Iran.
6. Rajaie Cardiovascular Medical and Research Center, Iran University of Medical Sciences, Tehran, Iran.
7. Department of Infectious Diseases and Tropical Medicine, Tehran University of Medical Sciences, Tehran, Iran

**Corresponding Author:** Dr Ehsan Mostafavi No. 69, Pasteur Ave., Department of Epidemiology and Biostatistics, Research Centre for Emerging and Reemerging Infectious Diseases, Pasteur Institute of Iran, Postal Code: 1316943551, Tehran, Iran, Telefax: +98-21-66496448, Email: [mostafavi@pasteur.ac.ir](mailto:mostafavi@pasteur.ac.ir), [mostafaviehsan@gmail.com](mailto:mostafaviehsan@gmail.com)

**Abstract**

Patients with the underlying valvular heart disease are at the high risk of developing sub-acute or chronic endocarditis secondary to *Coxiella burnetii*. Q fever endocarditis is the most common manifestation along with persistent the infection. There is some serologic and molecular evidence of *C. burnetii* infection in humans and livestock in Iran. As it is possible to observe chronic Q fever in Iran, it seems necessary to study the prevalence of Q fever endocarditis in this country. In the present study, Infective Endocarditis (IE) patients (possible or definite based on Duke Criteria) hospitalized in Rajaie Cardiovascular Medical and Research Center were enrolled from August 2016 to September 2018. Culture-negative endocarditis patients were evaluated by Raoult criteria for diagnosis Q fever endocarditis. The serological results for brucellosis were negative for all subjects. All blood and tissue samples including valve samples were tested for *C. burnetii* infection using serology and Polymerase Chain Reaction (PCR). In this study, 126 patients who were admitted to the hospital were enrolled; of which 52 subjects were culture-negative IE. Among the participants, 16 patients (30.77%) were diagnosed with Q fever IE and underwent medical treatment. The mean age of patients was 46.6 years ranging from 23 to 69 years and 75% of them were male. Considering the high prevalence of Q fever IE, evaluation of the patients with culture-negative IE for *C. burnetii* infections was highly recommended.

**Keywords:** Q fever, Endocarditis, *Coxiella burnetii,* Infective Endocarditis

## **Introduction**

Q fever is a zoonotic infection caused by an intracellular and pleomorphic gram-negative coccobacillus called *Coxiella burnetii* ^1^. It is also a spore-like formation that is highly resistant to osmotic, mechanical, chemical, heat, and desiccation stresses ^2^. This bacterium remains for about 7-10 months on wool in 15-20℃. It also stays alive on fresh meat in cold storage over a month and in milk or cream at room temperature in more than 40 months ^1^. *C. burnetii*, a bacterium that is the main cause of Q fever, has been identified in arthropods, fish, birds, rodents, and livestock. The most common reservoirs of this bacterium are cattle, goats, and sheep worldwide ^2^. Humans can be infected by Q fever through inhaling *C. burnetii* contained small aerosols ^3^.

Primary infections in humans can be symptomatic or asymptomatic ^4^. Therefore, patients with Q fever have an extensive spectrum of clinical presentations. Q fever is chiefly manifested as acute or chronic forms. The acute Q fever, the most prevalent type, is a self-limited febrile illness whose clinical manifestations include pneumonia, hepatitis, osteomyelitis, and cardiac involvement ^3^.

Chronic Q fever is very rare occurring in only 5% of patient’s e after the symptomatic or asymptomatic acute Q fever^2^. Clinical manifestations of chronic Q fever may include endocarditis, vasculitis, prosthetic joint arthritis, osteoarticular infection, and lymphadenitis ^1,5^. Endocarditis is the most prevalent form of chronic Q fever, involving 60-78% of all Q fever cases around the world. Infective endocarditis (IE) patients are predominantly men over the age of 40 ^3^. Although, Q fever endocarditis usually affects any other part of the vascular tree, prosthetic or abnormal native valves can be more infected ^6,7^. Valvular vegetation in Q fever endocarditis is usually very small and can be missed in echocardiography. Therefore, negative transesophageal echocardiogram (TEE) or transthoracic echocardiogram (TTE) does not exclude the diagnosis of Q fever ^3^. Mitral and aortic valves are the most commonly involved valves. The reports showed that about 10-19% of mortality rate was recorded in order to treat the lack of endocarditis Q fever^8^. Q fever endocarditis is presented as culture negative ^7,9^. The treatment for Q fever endocarditis is currently hydroxychloroquine and doxycycline ^10^. The treatment duration is 1.5 years for native valves and 2 years for prosthetic valves ^3^.

In 1952, the first acute Q fever cases were reported in Iran ^11^. In 1970, four cases were reported from Shiraz city, Iran ^12^. From 1970 to 1973, 45 acute Q fever cases were also reported from Abadan city (southwestern Iran). Moreover, 80 acute Q fever patients were diagnosed from 1972 to 1976. After 1976, the disease was forgotten for thirty-three years in Iran, and no human cases were reported ^11^. However, at the same time with a large outbreak of Q fever in Netherland, the investigation for Q fever was resumed in 2009. Since then, various seroepidemiological studies have been conducted on the animal and human population^13-18^. Human clinical cases of acute Q fever in Iran were re-reported ^19,20^. The first Q fever endocarditis in Iran was reported in Tehran in 2013 ^21^.

Since the epidemiology of Q fever varies worldwide and chronic Q fever, and that undiagnosed and untreated results in mortality may lead to culture-negative IE it seems necessary to study the prevalence of Q fever endocarditis in Iran.

## **Results**

Among 126 patients with IE (possible or definite based on Duke Criteria), were admitted in the hospital from August 2016 to September 2018, 52 subjects (36 males and 16 females) with culture-negative IE and the mean age (±SD) of 45.96 ± 17.78 years (ranged from 17 to 78) were enrolled in this study and 16 patients (30.77%) were diagnosed with Q fever IE and scheduled medical treatment. According to Raoult Criteria-based *C. burnetii* serology (with IFA), blood PCR, and heart valve tissue PCR (in the patients who haveundergone surgery), eleven patients with definite Q fever IE and five patients with possible Q fever IE were initially scheduled for treatments with hydroxychloroquine and doxycycline (Fig1).

The mean age of Q fever endocarditis patients was 46.6 years (ranged from 23 to 69). Among the sixteen patients diagnosed with Q fever, there were twelve men (75%) and four women (25%), of whom only one was living in rural areas (6.3%), but fourteen (87.5%) were using unpasteurized diaries; ten patients (62.5%) had a history of traveling to high-risk areas; and seven (43.8%) patients had contact with animals. None of the patients had a history of Q fever, tick bite, or immune defects. Fifteen patients (93.75%) had a history of valvular diseases, six patients (37.5%) had prosthetic valves, and two of whom (12.5%) presented with a history of endocarditis. Only one patient was diagnosed with the definite criteria of positive valvular PCR (6.3%), fifteen patients (93.7%) had positive serology, and one patient (6.3%) had positive blood PCR.

There was no report of tick bite and rash among the participants. Only valvular disease history was a significant risk factor for Q fever endocarditis (P= 0.016). No significant association was found between other studied variables and Q fever result in the culture-negative endocarditis cases. In the present study, 33.33% of men and 25% of women showed positive Q fever endocarditis. Although two subjects were referred from a rural area, one of whom showed positive Q fever endocarditis. Some participants with a history of unpasteurized dairy product diet also showed positive Q fever (Table 1).

Since Rajaie Cardiovascular Medical and Research Center in Tehran is a referral center, patients in our study had referred to the center from fifteen provinces of Iran. Most of the subjects were from Tehran (four patients), followed by Alborz, Khuzestan, and Qom provinces (two patients each).

Although all patients were fully informed on the dangers of their illness and were scheduled for treatment, one person refused to start treatment, two patients died before the treatment, and one patient died only about a month after receiving treatment (Table 2). The other twelve patients are still receiving treatment and are being frequently checked (every 6 months) by serological tests of IgG Phase I for *C. burnetii*. Based on fluorescent serological antibody tests, seven patients have significantly inhibited the titers of *C. burnetii*. All remaining twelve patients are receiving treatment, which was started initially with hydroxychloroquine and doxycycline; nonetheless, the treatment plan had to change to ciprofloxacin and doxycycline for two of the patients due to drug adverse reactions.

Among the eleven patients diagnosed with definite Q fever, seven patients were diagnosed with one major and three minor criteria, three patients with two major and two minor criteria, one patient with two major and three minor criteria, and one patient was diagnosed as definite by culture positive cardiac valve PCR. The remaining five patients were considered as possible Q fever by one major and two minor criteria.

**Discussions**

Among the 126 subjects of this study, 52 patients were culture-negative endocarditis, and among those 16 patients (30.77%) were diagnosed with Q fever endocarditis. The majority of the patients (96.15%) were living in the cities and they referred from fifteen provinces of Iran. Most subjects in our study were from Tehran, which is the capital city, and two of its neighboring cities (Karaj and Qom), which emphasizes the importance of considering Q fever endocarditis in culture-negative patients regardless of their place of living. As given in CDC guidelines, people who live around in a 17 km away from animals carrying *C. burnetii*, are more likely to be infected with this bacterium^3^. Although the direct contact with animals the main cause of the disease transmission^2^, there have been many other instances of Q fever affliction ^22,23^. In our study, more than half of the subjects diagnosed with Q fever endocarditis (56.2%) had no exposure to animals. A research in Spain showed that the vaccination of the cattle infected by *C. burnetii* did not completely eliminate the bacterium as it was still detectable ^24^.

Raw milk consumption can also be a less common cause of Q fever transmission to humans. In this study, 69.23% of subjects had a history of using unpasteurized dairy and 87.5% of positive Q fever patients had a history of unpasteurized dairy products diet. Although a history of exposure including contact with animals may contribute to the diagnosis of Q fever, no direct contact with animals might be neglected for the diagnosis or suspicion of the disease, because there is always a possibility of airborne transmission of *C. burnetii*, not to mention indirect contact with the infected animal can cause the Q fever outbreak. For instance in Switzerland, 350 people were infected by Q fever only because they lived alongside a road that was the sheep passage for the mountain pastures in Switzerland ^21^.

Q fever is an occupational disease that affects those who are in direct contact with the infected animals including ranchers, veterinarians, and workers of slaughterhouses^1,3^. Among the sixteen endocarditis Q fever patients in our study, only two were farmers and one patient was slaughterhouse worker.

To confirm the diagnosis of Q fever, the serological test in most cases is required. However, relying on the serological results is not enough; therefore, serological results must be interpreted by clinical judgment and other available information. In this study, the researchers practiced IFA (as a gold standard test) for the diagnosis of Q fever patients. Also, we used PCR for the diagnosis of DNA in clinical cases. According to our results, Q fever IE was diagnosed in sixteen cases. According to the Raoult Criteria-based *C. burnetii* serology (with IFA), blood PCR, and heart valve tissue PCR (in patients who have undergone surgery), eleven and five patients were respectively identified with definite and possible Q fever IE, respectively. Based on to Raoult criteria, IgG phase I ≥ 1:6400, blood PCR or positive blood culture was considered as the major criteria. Eventually 1:800 ≤ IgG Phase I and <1:6400 were considered as the minor criteria^5^.

Regarding the high prevalence of *C. burnetii* infection in culture-negative endocarditis patients, it is obvious that physician’s especially infectious disease specialists need to check *C. burnetii* infections in culture-negative endocarditis patients, because unsuccessful treatment of Q fever endocarditis associated with high mortality rates^25,26^. Since Rajaie Cardiovascular Medical and Research Center is a referral center and subjects were from different cities of Iran, this study demonstrated that *C. burnetii* infection is typical in different parts of Iran and must be seriously considered.

The design of this study was to evaluate the association between studied variables and Q fever IE among the culture-negative endocarditis cases. Since other causes of culture-negative endocarditis may also be zoonotic, the data presented in table 1 and later discussed, do not show whether or not these factors increase the risk for IE compared to the general population.

IFA is as a gold standard test for the diagnosis of Q fever patients. PCR can also be very helpful in the diagnosis of the disease, along with IFA. Based on valid documents, reported rates of PCR positivity in patients with Q fever endocarditis have almost 33%^25^. Unfortunately, most of the patients admitted to this study referred to Rajaie Cardiovascular Medical and Research Hospital, as the reference hospital for cardiovascular diseases in Iran, after receiving various and multiple treatments and being admitted to several hospitals. So, in this study, only 12.5% (two of 16) of Q fever endocarditis patients were PCR positive. Receiving a wide variety of antibiotics before (in other hospitals) and during the initial hospitalization in Rajaie Cardiovascular Medical and Research Hospital, could have a negative effect on the PCR result.

As it was discussed, the sampled hospital in this study is the national reference hospital for cardiovascular diseases, where patients with complex conditions are referred more, and the prevalence of Q fever endocarditis is predicted to be higher than other hospitals in Iran. It is recommended to do similar studies in other hospitals to clarify the real prevalence of this disease in the country.

This present study can be a big step to understanding of the high prevalence of this disease and the epidemiology of Q fever in Iran. Future studies should be conducted in this regard to shed light on the ways of prevention, diagnosis, and treatment of such infections with more number of subjects and other centers to hopefully increase the awareness among people, especially the medical staff who are dealing with this issue on a daily basis.

## **Methods**

This cross-sectional study was conducted in Rajaie Cardiovascular Medical and Research Center from August 2016 to September 2018. In this study, 126 possible IE or definite IE patients according to Duke Criteria were investigated^1,5,27^.Culture-negative endocarditis patients were evaluated by Raoult criteria for diagnosis Q fever endocarditis (Supplementary Table 1).

All stages of the study were approved by the research committee of the Rajaie Cardiovascular Medical and Research Center and the code of ethics was received from the ethical committee of this center (Code: RHC.AC.IR.REC.1395.48). Accordingly, all methods were performed in accordance with the institution’s relevant guidelines and regulations. All patients provided written informed consent. Researchers at all stages of the project were committed to protecting patients’ information. The research did not have any deleterious effects on the treatment of patients, nor did it impose any extra costs to the center or on patients. The two drugs used in the study, hydroxychloroquine and doxycycline, had no identified or uncontrolled side effects and were approved by the Food and Drug Administration (FDA).

After obtaining informed consent from participants, demographic characteristics, risk factors, and clinical symptoms were collected from each subject by a researcher-developed questionnaire. Then, a six ml blood sample for serum extraction and a three ml whole blood sample (contain EDTA) for molecular detection were taken from each patient. Also when there was a valve tissue specimen for the patients, the samples were sent to the laboratory for investigation. All samples were immediately transferred to the National Reference Laboratory for Plague, Tularemia, and Q fever in Research Centre for Emerging and Reemerging infectious diseases of Pasteur Institute of Iran.

Blood samples were centrifuged for 10 utesat 3000 rpm. After extraction of their sera, they were stored at -20℃ until the time of test. We used commercial IFA kit (Focus Diagnostics, Cypress, CA) for serologic diagnosis of Q fever according to the manufacturer’s instructions. IgG antibodies against both I and II phases antigens of *C. burnetii* were measured by IFA assay and anti-phase I IgG titers ≥1:800 for *C. burnetii* were considered positive ^4^.

For DNA extraction, 200 μL whole blood samples and 25 mg tissue specimens were employed. Genomic DNA was isolated using the QIAamp DNA Mini Kit (Qiagen, Germany) following the manufacturer’s “DNA purification from blood and tissue protocols”. Real-time PCR was performed using specific primers and probe sequences targeting the IS1111 gene of *C. burnetii*. To amplify the target gene, we used forward primer AAA ACG GAT AAA AAG AGT CTG TGG TT, reverse primer CCA CAC AAG CGC GAT TCA T, and probe 6-carboxyfluorescein (FAM) –AAA GCA CTC ATT GAG CGC G – TAMRA. Real-time PCR reactions were performed using the following reaction mixture: 12.5 μL of 2x RealQ Plus Master Mix for the probe (Ampliqon, Denmark), 900 nM forward primer, 900 nM reverse primer, 200 nM probe, and 5 μL of DNA template. Real-time was performed in the Corbett 6000 Rotor-Gene system (Corbett, Victoria, Australia) with a final volume of 25µL for each reaction. The PCR amplification program was performed in 10 minutes at 95℃, followed by 45 cycles of 15 s at 94℃ and 60 s at 60℃.

All data were imported to SPSS V.25.0.01 (IBM Corp. Released 2017. IBM SPSS Statistics for Windows, Version 25.0, Armonk, NY: IBM Corp.). Then, the mean, standard deviation, the range for quantitative variables, frequency, and percentage for qualitative variables were reported. Chi-squared and Fisher exact tests were used to compare the variables. A p-value less than 0.05 was considered statistically significant.

## **Acknowledgments**

The researchers would like to express our gratitude to Dr. Majid Kiavar, Dr. Kambiz Mozaffari, and Dr. Farzaneh Fotouhi in Rajaie Cardiovascular Medical and Research Center for their support in sampling.

**Financial support**

We would like to acknowledge Rajaie Cardiovascular Medical and Research Center (Grant No. 95066), Pasteur Institute of Iran and Centre for Communicable Disease in Ministry of Health (Grant No. 1181 and 810) for their financial support.

**Author Contributions**

P.M., S.E. and E.S. designed the study. P.M., M.K., M.M., A.S., H.R.P., S.B., N.N., B.G. and S.L. provided sampling data. S.E., M.R. and A.G. provided laboratory data. E.M., H.B.A. and F.B.A. analyzed the data. P.M., S.E., E.M., M.K., and S.A.D.M. drafted the paper. All authors reviewed and approved the final manuscript.

**Competing interests**

The authors of this article declare no conflicts of interest.

**Fig 1. Distribution of patients diagnosed with Q fever infective endocarditis (IE) according to Raoult criteria.**

**Table 1. Descriptive and analytic analysis of the association between studied variables and Q Fever result among the** **culture-negative endocarditis cases hospitalized in Rajaie Cardiovascular Medical and Research Center, Tehran, 2016- 2018.**

| **Variable** | **Category** | **Total**  **(N=52)** | **Positive for Q fever (%)** | **OR (95% CI)** | **P value** |
| --- | --- | --- | --- | --- | --- |
| Gender | Male | 36 | 12 (33.33) | 0.67 (0.18-2.51) | 0.55 |
|  | Female | 16 | 4 (25.00) |  |  |
| Age (Median=44.5 Year) | <44.5 Years | 26 | 9 (34.62) | 0.70 (0.21-2.28) | 0.55 |
|  | ≥ 44.5 years | 26 | 7(26.92) |  |  |
| Living area | Urban | 50 | 15 (30.00) | 2.33 (0.14-39.82) | 0.53 |
|  | Rural | 2 | 1 (50.00) |  |  |
| History of drinking of unpasteurized dairy product | No | 16 | 2 (12.50) | 4.46 (0.88-22.65) | 0.10 |
|  | Yes | 36 | 14 (38.89) |  |  |
| History of contact with animal | No | 27 | 9 (33.33) | 0.78( 0.24- 2.54) | 0.68 |
|  | Yes | 25 | 7 (28.00) |  |  |
| History of artificial heart valve | No | 30 | 10 (33.33) | 0.75 (0.21-2.55) | 0.65 |
|  | Yes | 22 | 6 (27.27) |  |  |
| History of previous endocarditis | No | 47 | 14 (29.79) | 1.57 (0.24-10.46) | 0.64 |
|  | Yes | 5 | 2 (40.00) |  |  |
| History of Immune system deficiency | No | 50 | 16 (32.00) | - | 0.99 |
|  | Yes | 2 | 0 (0.00) |  |  |
| History of valvular disease | No | 15 | 1 (6.67) | 9.23 (1.39-216.4) | 0.016 |
|  | Yes | 37 | 15(40.54) |  |  |
| Trip history | No | 28 | 6 (21.42) | 2.62 (0.78-8.82) | 0.12 |
|  | Yes | 24 | 10 (41.67) |  |  |
| Fever | No | 11 | 4 (36.36) | 0.84 (0.21-3.43) | 0.99 |
|  | Yes | 37 | 12 (32.43) |  |  |
| Myalgia | No | 27 | 10 (37.04) | 0.68 (0.20-2.32) | 0.54 |
|  | Yes | 21 | 6 (28.57) |  |  |
| Chest pain | No | 30 | 10 (33.33) | 1.00 (0.29-3.45 | 0.99 |
|  | Yes | 18 | 6 (33.33) |  |  |
| Night sweats | No | 23 | 8 (34.78) | 0.88 (0.27-2.93) | 0.83 |
|  | Yes | 25 | 8 (32.00) |  |  |
| Fatigue | No | 17 | 5 (29.41) | 1.32 (0.37-4.73) | 0.67 |
|  | Yes | 31 | 11 (35.48) |  |  |
| Cough | No | 32 | 12 (37.50) | 0.56 (0.15- 2.12) | 0.39 |
|  | Yes | 16 | 4 (25.00) |  |  |
| Headache | No | 30 | 13 (43.33) | 0.26 (0.06-1.10) | 0.07 |
|  | Yes | 18 | 3 (16.67) |  |  |
| Splenomegaly | No | 47 | 16 (34.04) | - | 0.99 |
|  | Yes | 1 | 0 (0.00) |  |  |
| Hepatomegaly | No | 47 | 15 (31.91) | - | 0.33 |
|  | Yes | 1 | 1 (100.00) |  |  |
| Chronic pneumonia | No | 45 | 15 (33.33) | 1.00 (0.08-11.93) | 0.99 |
|  | Yes | 3 | 1 (33.33) |  |  |
| Shortness of breath | No | 45 | 15 (33.33) | 1.00 (0.08-11.93) | 0.99 |
|  | Yes | 3 | 1 (33.33) |  |  |
| Echocardiographic evidence | No | 2 | 0 (0.00) | - | 0.99 |
|  | Yes | 50 | 16 (32.00) |  |  |

**Table 2. Epidemiological factors, laboratory and clinical findings of patients with Q fever endocarditis hospitalized in Rajaie Cardiovascular Medical and Research Center, Tehran, 2016- 2018.**

| **No.** | **Age/Sex** | **Residency/ job** | **Animal Keeping History** | **Prosthetic Valve or** Cardiovascular **Implantable Electronic Device** | **ESR** | **Clinical Signe** | **Echocardiography Findings** | **Titers of IgG (IFA)** | | **PCR** | **Diagnosis of Q Fever Endocarditis (Possible or Definitive)** | **Outcome** |
| --- | --- | --- | --- | --- | --- | --- | --- | --- | --- | --- | --- | --- |
|  |  |  |  |  |  |  |  | I | II |  |  |  |
| **1** | 59/F | Urban/Housekeeper | Yes (Brid) | No | 83 | Fatigue, Cough, Weight loss | Vegetation (Mitral Valve) | 1024 | 2048 | Negative | Possible | Follow-up |
| **2** | 37/M | Urban/Mining engineer | Yes (Dog) | Yes(Pulmonary Valve) | 94 | Fever, Night sweats, Chronic pneumonia, Headache, Fatigue, | Vegetation (Pulmonary Valve) | 131072 | 65536 | Negative | Definitive | Lack of follow-up |
| **3** | 33/M | Urban/Photographer | No | Yes( Pulmonary Valve) | 45 | Cough, Fatigue, Fever, , Night sweats | Vegetation (Pulmonary Valve) | 32768 | 16384 | Negative | Definitive | Follow-up |
| **4** | 69/M | Urban/ Retired employee | No | Yes (ICD) | 90 | Cough, Fatigue, Fever, Night sweats, Myalgia | Vegetation (ICD lead Pace) | 1024 | 512 | Negative | Definitive | Died |
| **5** | 23/F | Urban/Housekeeper | Yes (Brid) | Yes(Pulmonary Valve) | 90 | Cough, Fatigue, Fever, Chest pain, Weight loss, Anemia, Anorexia | Vegetation (Pulmonary valve) | 2048 | 2048 | Negative | Definitive | Follow-up |
| **6** | 25/M | Urban/Slaughterhouse worker | Yes (Brid, Sheep and Goat) | No | 90 | Chest pain, Fever, Night sweats, Myalgia | Vegetation (Pulmonary Valve) | 2048 | 2048 | Negative | Definitive | Follow-up |
| **7** | 44/M | Urban/Driver | Yes (Bird, Cow, Sheep and Goat) | No | 12 | Anemia, Anorexia | Vegetation (Aortic Valve) | 4096 | 4096 | Negative | Possible | Follow-up |
| **8** | 54/F | Urban/Housekeeper | No | Yes (Aortic and Mitral Valves) | 19 | Myalgia, Fatigue, Fever, Chest pain, Weight loss, Anemia, Anorexia | Vegetation (Repaired Tricuspid) | 4096 | 4096 | Negative | Possible | Lack of follow-up |
| **9** | 33/F | Urban/Housekeeper | No | No | 45 | Myalgia, Fatigue, Fever, Chest pain | Severe Mitral Regurgitation | 32768 | 32768 | Negative | Possible | Follow-up |
| **10** | 58/M | Rural/Farmer | Yes (Cow, Sheep and Goat) | No | 45 | Fatigue, Myalgia, Headache, Hepatosplenomegaly, Anemia, Anorexia | Vegetation (Aortic Valve) | 1024 | 1024 | Positive (Blood) | Definitive | Follow-up |
| **11** | 56/M | Urban/Retired employee | No | No | 25 | Fever, Night sweats , Anemia, Weight loss | Vegetation (Mitral Valve) | 4096 | 2048 | Negative | Definitive | Follow-up |
| **12** | 29/M | Urban/Welding worker | No | No | 64 | Chest pain, Fever, , Night sweats, Myalgia, Headache, Weight loss, Anorexia | Vegetation (Mitral and Aortic Valve ) | 8192 | 8192 | Negative | Definitive | Follow-up |
| **13** | 60/M | Urban/Farmer | Yes (Brid, Cat, Dog, Cow, Sheep and Goat) | Yes(Aortic and Mitral Valves) | 33 | Chest pain, Night sweats | Vegetation (Mitral) | 2048 | 512 | Negative | Possible | Died |
| **14** | 40/M | Urban/ Supermarket worker | No | No | 25 | Fever | Vegetation (Tricuspid and Aortic Valves ) | 256 | 512 | Positive (Valve) | Definitive | Died |
| **15** | 67/M | Urban/Teacher | No | No | 3 | Myalgia, Fatigue, Fever | Vegetation (Mitral Valve ) | 1024 | 1024 | Negative | Definitive | Follow-up |
| **16** | 26/M | Urban/Student | No | Yes(Aortic Valve) | 4 | Fatigue, Night sweats , Fever | Pseudoaneurysm of aorta , Dehiscence | 4096 | 8192 | Negative | Definitive | Follow-up |

**Supplementary Table 1. Diagnostic guideline for Q fever endocarditis proposed by Raoult criteria^25^.**

**References**

1 Eldin, C. *et al.* From Q fever to Coxiella burnetii infection: a paradigm change. *Clinical microbiology reviews* **30**, 115-190 (2017).

2 Angelakis, E. & Raoult. Q fever. *Veterinary Microbiology* **140**, 297–309 (2010).

3 Anderson, A. *et al.* Diagnosis and management of Q fever--United States, 2013. *MMWR. Recommendations and reports: Morbidity and mortality weekly report. Recommendations and reports/Centers for Disease Control* **62**, 1-30 (2013).

4 Raoult, D., Marrie, T. & Mege, J. Natural history and pathophysiology of Q fever. *The Lancet infectious diseases* **5**, 219-226 (2005).

5 Raoult, D. Chronic Q fever: expert opinion versus literature analysis and consensus. *Journal of Infection* **65**, 102-108 (2012).

6 Grisoli, D. *et al.* Latent Q fever endocarditis in patients undergoing routine valve surgery. *The Journal of heart valve disease* **23**, 735-743 (2014).

7 Wegdam-Blans, M. C. *et al.* Chronic Q fever: review of the literature and a proposal of new diagnostic criteria. *Journal of Infection* **64**, 247-259 (2012).

8 Fenollar, F., Thuny, F., Xeridat, B., Lepidi, H. & Raoult, D. Endocarditis after acute Q fever in patients with previously undiagnosed valvulopathies. *Clinical infectious diseases* **42**, 818-821 (2006).

9 Kampschreur, L. M. *et al.* Identification of risk factors for chronic Q fever, the Netherlands. *Emerging infectious diseases* **18**, 563-570 (2012).

10 Gould, F. K. *et al.* Guidelines for the diagnosis and antibiotic treatment of endocarditis in adults: a report of the Working Party of the British Society for Antimicrobial Chemotherapy. *Journal of antimicrobial chemotherapy* **67**, 269-289 (2012).

11 Mostafavi, E., Rastad, H. & Khalili, M. Q Fever: An emerging public health concern in Iran. *Asian Journal of Epidemiology* **5**, 66-74 (2012).

12 Eghtedari, A., Kohout, J. & Path, M. Q fever in Iran A report of clinical cases and serological studies in Shiraz. *Pahlavi Med J* **1**, 66-73 (1970).

13 Esmaeili, S., Pourhossein, B., Gouya, M. M., Amiri, F. B. & Mostafavi, E. Seroepidemiological survey of Q fever and brucellosis in Kurdistan Province, western Iran. *Vector-Borne and Zoonotic Diseases* **14**, 41-45 (2014).

14 Esmaeili, S., Bagheri Amiri, F. & Mostafavi, E. Seroprevalence survey of Q fever among sheep in northwestern Iran. *Vector-Borne and Zoonotic Diseases* **14**, 189-192 (2014).

15 Esmaeili, S., Mostafavi, E., Shahdordizadeh, M. & Mahmoudi, H. A seroepidemiological survey of Q fever among sheep in Mazandaran province, northern Iran. *Annals of agricultural and environmental medicine: AAEM* **20**, 708-710 (2013).

16 Kayedi, M. H. *et al.* Seroepidemiological study of Q fever in Lorestan province, western Iran, 2014. *Iranian journal of microbiology* **9**, 213-218 (2017).

17 Mobarez, A. M., Amiri, F. B. & Esmaeili, S. Seroprevalence of Q fever among human and animal in Iran; A systematic review and meta-analysis. *PLoS neglected tropical diseases* **11**, e0005521 (2017).

18 Esmaeili, S. *et al.* Seroprevalence of brucellosis, leptospirosis, and Q fever among butchers and slaughterhouse workers in south-eastern Iran. *PloS one* **11**, e0144953 (2016).

19 Esmaeili, S., Golzar, F., Ayubi, E., Naghili, B. & Mostafavi, E. Acute Q fever in febrile patients in northwestern of Iran. *PLOS Neglected Tropical Diseases* **11**, e0005535 (2017).

20 Khalili, M., Reza Naderi, H., Salehnia, N. & Abiri, Z. Detection of Coxiella burnetii in acute undifferentiated febrile illnesses (AUFIs) in Iran. *Tropical doctor* **46**, 221-224 (2016).

21 Yaghmaie, F., Esmaeili, S., Francis, S. A. & Mostafavi, E. Q fever endocarditis in Iran: A case report. *Journal of infection and public health* **8**, 498-501 (2015).

22 Das, I., Steeds, R. & Hewins, P. Chronic Q fever: An ongoing challenge in diagnosis and management. *Canadian Journal of Infectious Diseases and Medical Microbiology* **25**, 35-37 (2014).

23 Marrie, T. J. & Raoult, D. Q fever—a review and issues for the next century. *International journal of antimicrobial agents* **8**, 145-161 (1997).

24 Astobiza, I. *et al.* A four-year evaluation of the effect of vaccination against Coxiella burnetii on reducing animal infection and environmental contamination in a naturally infected dairy sheep flock. *Applied and environmental microbiology*, AEM. 05530-05511 (2011).

25 Million, M., Thuny, F., Richet, H. & Raoult, D. Long-term outcome of Q fever endocarditis: a 26-year personal survey. *The Lancet infectious diseases* **10**, 527-535 (2010).

26 Straily, A., Dahlgren, F. S., Peterson, A. & Paddock, C. D. Surveillance for Q Fever Endocarditis in the United States, 1999–2015. *Clinical Infectious Diseases* **65**, 1872-1877 (2017).

27 Li, J. S. *et al.* Proposed modifications to the Duke criteria for the diagnosis of infective endocarditis. *Clinical infectious diseases* **30**, 633-638 (2000).
